# Potentiation of paclitaxel-induced apoptosis in a non-small cell lung cancer model using the traditional Chinese drug huaier: Network pharmacology analysis, experimental verification, and clinical impact 

**DOI:** 10.5414/cp204745

**Published:** 2025-05-13

**Authors:** Wanrong Zheng, Fobao Lai

**Affiliations:** 1 College of Medical Nursing, Minxi Vocational and Technical College, and; 2 Department of Oncology, Longyan First Affiliated Hospital of Fujian Medical University, Longyan, China

**Keywords:** Huaier, paclitaxel, network pharmacology, non-small-cell lung cancer

Sir, – The effectiveness of paclitaxel (PTX) against non-small cell lung cancer (NSCLC) is limited [1]. Huaier has a notable anticancer effect on lung carcinoma; however, the specific mechanism remains unknown, and the potential effects of combining huaier with PTX for NSCLC treatment are still uncertain. 

The active ingredients of huaier were identified by the Batman and Herb database, including genistein, kaempferol, rutin, and glucuronic acid. Furthermore, the SwissTargets database was employed to identify the targets of these active ingredients and PTX. After deweighting and correction, 312 targets were obtained from UniProt. The GeneCards, National Center for Biotechnology Information (NCBI), and Comparative Toxicogenomics Database (CTD) databases were searched for NSCLC-related genes. Following the removal of duplicate genes, disease-related genes from the three databases were selected for further analysis. Furthermore, an updated Venn diagram based on chosen disease and medication targets was created via software and revealed 134 common targets that act as predicted drug action targets in diseases. GO and KEGG analysis were performed on 134 targets related to PTX, huaier, and NSCLC. KEGG analysis identified the top 20 pathways with BH-corrected p-values < 0.05. The main components of these pathways were the PI3K-AKT signaling pathway, cancer-related microRNAs, cancer-involved proteoglycans, receptor activation in chemical carcinogenesis, and cellular senescence. 

The main components of huaier were determined by conducting a topological examination of component-disease-target networks, which were established using Cytoscape 3.8.0 software based on their degree values. Components with higher scores, indicating greater importance, are prioritized for future research. Genistein was identified as the most valuable compound, followed by paclitaxel, kaempferol, rutin, and glucuronic acid. 

The Cytoscape 3.8.0 program was used to construct a network of ingredient‒disease pathway targets (Figure 1A). The network comprised 134 targets associated with NSCLC-related diseases (excluding free protein spots), 5 active ingredients (excluding ingredients not directly linked to the targets), and 20 pathways related to NSCLC targets. Among all the active compounds, genistein presented the greatest number of linked edges. The linked edges of TP53, AKT1, EGFR, HSP90AA1, and VEGFA were higher than those of other possible targets. The PI3K-AKT signaling pathway, cancer-associated microRNAs, cancer-associated proteoglycans, receptor activation in chemical carcinogenesis, and cellular senescence, all of which are among these pathways, showed a higher number of links with possible targets. 

An Annexin V-FITC Apoptosis Detection Kit (Multi Sciences Biotech, Hangzhou, China) was utilized to assay apoptosis. Figure 1B and C show that the co-administration of PTX (1 µM) and huaier (1 mg/mL) effectively induce H1299 cell apoptosis. The apoptosis rate with the combination treatment was 60.54%, which was significantly greater than that with PTX alone (20.46%) or huaier alone (14.03%). An assessment of their effect on lung cancer cell proliferation was performed using a CCK8 assay. The illustration in Figure 1C reveals that huaier (1 mg/mL) and PTX (1 µM) reduced the growth of H1299 cells compared to the control group. What’s more, the group that received a combination of treatments exhibited a notably diminished rate of proliferation. 

To examine the interaction of the huaier active ingredient and PTX with the EGFR and AKT1 proteins, we performed computational docking analysis. The receptor proteins EGFR and AKT1 were extracted from the pdb database. The receptor protein was dewatered and deligand using PYMOL 2.3.4 software, while AutoDockTools software was employed to hydrogenate and equalize the receptor protein‘s charge. Additionally, the receptor protein and ligand small molecules were transformed into pdbqt formats [2]. Molecular docking of receptor proteins and ligand small molecules [3] was conducted using AutoDock Vina 1.1.2, with protein-ligand interaction profiler (PLIP) [4] employed for the analysis of the docking outcomes. Molecular docking is carried out using AutoDockTools 1.1.2, where the binding energy magnitude suggests the probability of receptor-ligand binding. A lower binding energy indicates a higher affinity between the receptor and the ligand as well as a more stable conformation. The binding energy of molecular docking is collected in AutoDockTools 1.1.2. 

The results of the docking process indicated that these compounds could effectively bind to the protein kinase domains of EGFR and AKT1 through multiple interactions. The molecular docking binding energies for these sites are summarized in Table 1. It is evident that the active components of huaier and PTX can inhibit the activities of EGFR and AKT1 kinases, thereby suppressing EGFR/AKT signaling. 

The molecular mechanisms behind the antitumor impact of PTX and huaier on NSCLC were explored by utilizing real-time PCR to identify the expression of AKT1 and EGFR. PTX and huaier significantly reduced AKT1 and EGFR mRNA expression levels, particularly in the combined treatment group (Figure 1D, E). 

Numerous studies suggest that the EGFR/PI3K/AKT pathway is essential in enhancing cell growth, epithelial-mesenchymal transition (EMT), and metastasis [5]. Cyclin D1, a vital controller of the G1 checkpoint transition during the cell cycle, is associated with nuclear EGFR. This is activated by chemotherapy in cells with high proliferation, leading to its translocation [6]. Matrix metalloproteinases (MMPs), which are important regulators of cell motility and invasion, are also produced in greater quantities and with greater activity due to EGFR-dependent ERK and AKT activation [7]. Our research results indicate that the combination of PTX and huaier successfully suppresses the growth of NSCLC cells by reducing the levels of EGFR and AKT, aligning with prior studies [8]. 

The combination of huaier and PTX significantly inhibited the development of NSCLC, enhancing the efficacy of the treatment. Moreover, combination therapy with huaier and PTX has the potential to reduce the dosage of paclitaxel and minimize its toxic side effects. This is highly important, as high doses of paclitaxel can lead to severe side effects such as neurotoxicity [9] and bone marrow suppression [10]. By lowering the dose of paclitaxel, we can potentially increase the tolerability and safety of the treatment. However, combinations of multiple drugs may act synergistically by binding to multiple or the same target proteins [11]; the mechanism of huaier in combination with PTX and the optimal concentration ratio of huaier and PTX need to be investigated in vitro and in vivo. 

## Conclusion 

The compounds and targets exhibit a strong binding activity, as suggested by their high binding energy scores. Moreover, the combination of PTX and huaier exhibited a cooperative impact on promoting apoptosis and restraining the growth of NSCLC cells. The research showed a notable reduction in the expression levels of AKT and EGFR mRNA after being exposed to PTX and huaier. Conclusion: The findings imply that the active elements in huaier could potentially work in conjunction with PTX to target EGFR and AKT, thereby inhibiting the proliferation of NSCLC cells. 

## Data availability statement 

The authors will make the raw data supporting the conclusions of this article available without any reservations. 

## Authors’ contributions 

Besides writing the manuscript, Wanrong Zheng analyzed the data and designed the study. Fobao Lai implemented, conducted, and analyzed the study. All authors approved the final version of the manuscript. 

## Funding 

Sponsored by The Education and Scientific Research Project for Middle-Aged and Young Teachers in Fujian Province (JAT220750) and Fujian Province Natural Science Foundation (grant number: 2023J011900). 

## Conflict of interest 

The authors declare that they have no conflict of interest. 

**Figure 1:**
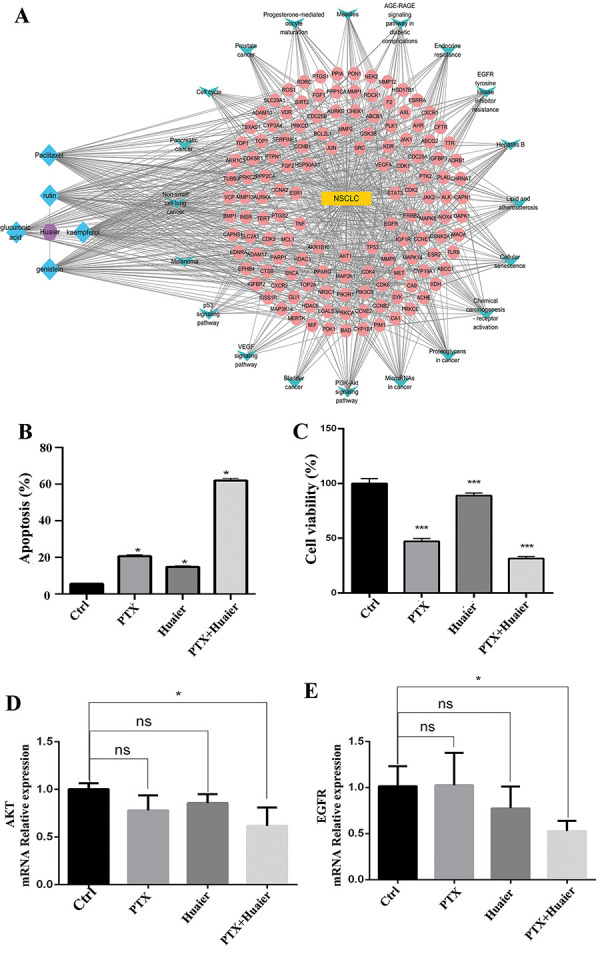
The synergistic efficacy of Huaier in combination with paclitaxel against non-small cell lung cancer (NSCLC). A: Network diagram illustrating the relationship between drug components, targets, and pathways. B: H1299 cells were treated with PTX (1uM), huaier (1 mg/mL), or their combinations for 48 hours. Apoptosis was measured using flow cytometry. C: A cell viability assay was performed by CCK8. D, E: Real-time PCR array was used to measure the levels of AKT and EGFR mRNA expression in H1299 cells. The data is presented as the average plus or minus the standard deviation (n = 3). *p < 0.05, **p < 0.01,***p < 0.001.

**Table 1.** Molecular docking data of huaier active ingredients and paclitaxel with proteins AKT1 and EGFR.

	AKT1	EGFR
Genistein	–7.7	–8.3
Glucuronicacid	–5.6	–5.7
Kaempferol	–8.1	–8.5
Paclitaxel	–7.9	–7.9
Rutin	–8.9	–9.7
